# PHAGE-2 Study: Supplemental Bacteriophages Extend *Bifidobacterium animalis* subsp. *lactis* BL04 Benefits on Gut Health and Microbiota in Healthy Adults

**DOI:** 10.3390/nu12082474

**Published:** 2020-08-17

**Authors:** Diana S. Grubb, Scott D. Wrigley, Kimberley E. Freedman, Yuren Wei, Allegra R. Vazquez, Roxanne E. Trotter, Taylor C. Wallace, Sarah A. Johnson, Tiffany L. Weir

**Affiliations:** 1 Intestinal Health Laboratory, Department of Food Science and Human Nutrition, Colorado State University, Fort Collins, CO 80523, USA; Diana.Grubb@colostate.edu (D.S.G.); Scott.Wrigley@colostate.edu (S.D.W.); Kim.Freedman@colostate.edu (K.E.F.); Yuren.Wei@colostate.edu (Y.W.); Allegra.Stroud@colostate.edu (A.R.V.); Roxytrot@gmail.comz (R.E.T.); 2 Functional Foods & Human Health Laboratory, Department of Food Science and Human Nutrition, Colorado State University, Fort Collins, CO 80523, USA; Sarah.Johnson@colostate.edu; 3 Think Healthy Group, 1301 20th Street, NW, #413, Washington, DC 20036, USA; taylor.wallace@me.com; 4 Department of Nutrition and Food Studies, George Mason University, 4400 University Drive, MS:1F7, Fairfax, VA 22030, USA

**Keywords:** bacteriophage, *Bifidobacterium*, gut microbiota, intestinal health, microbiome, probiotic

## Abstract

Probiotics are increasingly used by consumers and practitioners to reduce gastrointestinal (GI) distress and improve gut function. Here, we sought to determine whether the addition of supplemental bacteriophages (PreforPro) could enhance the effects of a common probiotic, *Bifidobacterium animalis* subsp. *lactis* (*B. lactis*) on GI health. A total of 68 participants were enrolled in a 4-week, randomized, parallel-arm, double-blind, placebo-controlled trial where primary outcomes included self-assessments of GI health, a daily stool log, and 16s rRNA analysis of gut microbial populations. We observed within-group improvements in GI inflammation (*p* = 0.01) and a trending improvement in colon pain (*p* = 0.08) in individuals consuming *B. lactis* with PreforPro, but not in the group consuming only the probiotic. There was also a larger increase in *Lactobacillus* and short-chain fatty acid-producing microbial taxa detected in the stool of participants taking PreforPro with *B. lactis* compared to the probiotic alone. Overall, these results suggest the addition of PreforPro as a combination therapy may alter gut ecology to extend the GI benefits of consuming *B. lactis* or other probiotics.

## 1. Introduction

Over the past two decades, research on the gut microbiota has revealed that it plays an important role in maintaining health and preventing disease [1]. Although novel clinical applications of microbiota modulation are mainly restricted to fecal microbiota transplantation for treatment of *Clostridium difficile* [2], gut-targeted dietary supplements are a large and growing segment of the public market [3] Among these dietary supplements are probiotics, live beneficial microorganisms that confer benefits to the host through a variety of mechanisms [4]. In addition to commercial availability as over-the-counter dietary supplements, they can also be found as natural components in many fermented foods or supplemented additives in non-fermented food products. Probiotics are being explored as an accessible, convenient, and relatively low-cost strategy to modify the gut microbiota for a variety of human health outcomes ranging from improved gastrointestinal (GI) function to the management of lipid and glucose metabolism [5]. While there is still minimal scientific evidence to support many of these uses, there are data supporting the efficacy of probiotics for improving GI function. Efficacy appears to be somewhat dependent on the specific species/strains, or the combination, used for a given condition; however, a 2012 meta-analysis of randomized controlled trials showed that 8 of the 11 species/mixtures tested were efficacious for a range of GI conditions, including pouchitis, infectious and *C. difficile* associated-diarrhea, Irritable Bowel Syndrome (IBS), and antibiotic-associated diarrhea [6]. A more recent meta-analysis suggests that probiotics may also be beneficial in alleviating functional constipation in adults [7]. Even in cases where there were insufficient data to demonstrate probiotic efficacy, adverse effects were minimal [8], suggesting probiotics are a low-risk intervention to improve GI health in humans.

Bacteriophages, or phages, are also being explored for their potential to selectively modify the gut microbiota. Phages attach to a specific host bacterium, insert their genetic material into the cell, and take over the machinery of the host cell to replicate phage components. Once this process is complete, the cell is destroyed, or lysed, releasing new phage particles. Unlike antibiotics, phages display a narrow host range, limiting global perturbations to the gut microbiota that can promote dysbiosis, reduce intestinal homeostasis, and promote disease. In addition to their narrow host range, phages are ubiquitous in the environment, and many are Generally Regarded as Safe (GRAS) for human consumption. We recently showed that supplemental consumption of a cocktail of *E. coli*-targeting bacteriophages, marketed as PreforPro, was considered safe and tolerable [9]. We also demonstrated that these phages showed bifidogenic effects after 4 weeks of oral supplementation, with no global disruption of the gut microbiota [10]. Based on these data, we hypothesized that, in addition to reducing target host species, phages may also reduce competition among commensal bacteria for limited resources and produce a potential fuel source in the form of contents released from the dead bacteria. Therefore, phages taken in combination with a probiotic may extend benefits on GI health and intestinal environment.

In the current study, we tested whether a combined probiotic and phage-based intervention extends probiotic impacts on gastrointestinal discomfort and stool consistency in a healthy adult population. Our randomized, double-blind, placebo-controlled intervention included three parallel arms in which participants were assigned to consume either *Bifidobacterium animalis* subsp. *lactis* BL04 (*B. lactis*), *B. lactis* with a commercial phage cocktail targeting *E. coli*, or a maltodextrin-based placebo. We selected this combination because our previous data showed that PreforPro increased commensal *Bifidobacterium* [10]. The primary outcome measures included subjective digestive health questionnaires and stool consistency measurements based on the Bristol Stool Scale. We hypothesized that probiotic-consumption would improve one or more aspects of digestive health and stool consistency in our participant population and that addition of the phage cocktail would potentiate these effects. Specifically, our primary outcome measures included a digestive health questionnaire, stool consistency measurements, and molecular analysis of the gut microbiota. We hypothesized that consumption (*B. lactis* BL04 + PreforPro) would improve one or more aspects of digestive health and stool consistency in our participant population compared to the probiotic alone.

## 2. Materials and Methods

### 2.1. Study Design

The BacterioPHAGE for Gastrointestinal Health 2 Study (PHAGE-2 Study; #NCT04511221) is a 4-week, randomized, parallel-arm, double-blind, placebo-controlled clinical intervention trial. The PHAGE 2 Study was designed to test whether combining supplemental bacteriophages with a probiotic would provide additional benefits to GI health and gut microbiota compared to consuming a probiotic alone. Data from our previous study [10] were used to determine sample size, based on 80% power to detect a significant change (alpha = 0.05) in *Bifidobacterium* levels between the placebo and treatment groups. The Colorado State University Institutional Review Board approved the study protocol (CSU #19-9145H) and all 68 participants enrolled in one of the three study arms provided written informed consent. This trial is registered at ClinicalTrials.gov under #NCT04511221.

Participants were pre-qualified by phone screening and attended an in-person study visit at Colorado State University’s Food and Nutrition Clinical Research Laboratory (FNCRL) in the Department of Food Science and Human Nutrition. Participants were fasted at least 8 h and asked to abstain from exercise for at least 12 h prior to clinic visits. They were also instructed to refrain from taking any medications or dietary supplements for 24 h prior to their visits. During their initial visit (baseline), eligibility was confirmed by taking anthropometric measurements of height (cm) and weight (kg) to calculate BMI and completing a written medical health questionnaire to determine medical history and current medication use. At both baseline and 4-week (final) visits, participants were asked to complete a digestive health questionnaire, undergo several measures of cardiovascular function (reported in Trotter et al. 2020 in press), and provide a venous blood and stool sample. They were also provided with a stool log and asked to keep a daily record of their bowel movements.

After undergoing sample collections and analysis procedures at the baseline visit, participants were randomly assigned to 1 of 3 treatments groups: (A) 15 mg rice maltodextrin (placebo); (B) 1 × 10^9^ Colony Forming Units (CFU) *Bifidobacterium animalis* subspecies *lactis* strain BL04 (*B. lactis* BL04); or (C) 1 × 10^9^ CFU *B. lactis* BL04 + 1 × 10^6^ Plaque Forming Units (PFU) LH01-Myoviridae, LL5-Siphoviridae, T4D-Myoviridae, and LL12-Myoviridae bacteriophages, marketed as PreforPro (*B. lactis* BL04 + PreforPro). Participants were asked to consume one 15-mg capsule per day during the 4-week intervention period. All treatment capsules contained maltodextrin from rice and medium chain triglycerides from palm and coconut oil as a filler material. Participants were randomized to treatment groups using the second generator at www.randomization.com. To assess treatment compliance, participants returned all unused treatment capsules at their final visit.

Participants were asked to maintain their normal eating and exercise habits throughout the course of the study. To aid in determining dietary compliance, participants were completed a 2-day diet record at the beginning and end of the treatment period, including 1 weekday and 1 weekend day, using the National Institutes of Health, National Cancer Institute Automated Self-Administered 24-h dietary assessment tool (ASA24; https://asa24.nci.nih.gov). For each of the 2-day diet records, participants recorded all foods, drinks, and supplements they consumed in a 24-h period and the time of day they were eating and drinking.

### 2.2. Participant Characteristics

Healthy, adult participants were recruited from Fort Collins, Colorado and surrounding areas by referral from local healthcare practitioners, flyers, email, and through word of mouth. Initial eligibility was determined by a phone screening questionnaire and confirmed onsite at the FNCRL via interview and BMI assessment by the clinical coordinator. Inclusion and exclusion criteria are presented in Table 1. Participants were asked to maintain their regular diet and exercise habits as well as to limit alcohol consumption to 1–2 drinks per day or no more than 8 drinks per week during the study. They were also asked to abstain from taking any supplemental pre- or probiotics. A summary of baseline participant characteristics can be found in Table 2.

### 2.3. Comprehensive Metabolic Panels

Blood samples (100 uL) were collected from the antecubital vein in a lithium heparin tube and immediately analyzed using a Piccolo Metlyte Plus CRP Reagent Disc, which included glucose, blood urea nitrogen (BUN), creatinine (CRE), creatinine kinase (CK), NA+, K+, Cl- and C-reactive protein (CRP), on the Piccolo Xpress Chemistry Blood Analyzer (Abaxis, Union City, CA, USA).

### 2.4. Digestive Health and Bowel Movement Assessment

Participants completed a digestive health questionnaire at baseline and after 4 weeks on treatment to assess perceived effects on GI symptoms, as previously described [9]. Briefly, the questionnaire had 4 sections corresponding to gastric function, GI inflammation, small intestine (SI) and pancreas pain, and colon function (Supplemental Methods File S1). Participants ranked questions within each section, choosing no/rarely, occasionally, often, or frequently. Symptom severity was then ranked according to their priority status outlined in Table 3. The percentage of participants that experienced low, moderate, and high priority gastrointestinal symptoms at the initial and follow-up visit were calculated. Initial test scores (baseline) and retest scores (week 4) were compared within treatment groups across sections of the questionnaire to assess the percentage of participants that experienced an improvement, worsening, or no change to the severity of their gastrointestinal symptoms after the 4-week intervention.

Participants were also asked to record all bowel movements during the study and rank them according to the Bristol Stool chart. Participants were given a copy of the Bristol Stool Chart as a reference to help determine stool type. Types included Type 1 (separate hard lumps), Type 2 (lumpy and sausage like), Type 3 (a sausage shape with cracks in the surface), Type 4 (like a smooth, soft sausage or snake), Type 5 (soft blobs with clear-cut edges), Type 6 (mushy consistency with ragged edges) and Type 7 (liquid consistency with no solid pieces). For data analysis, each stool type was coded as either hard (Types 1 or 2), soft (Types 3, 4, or 5), or diarrhea (Type 6 or 7). Stools categorized as soft were considered normal, while hard and diarrhea stools were considered abnormal.

### 2.5. Stool Sample Processing

Stool samples were collected at home, stored refrigerated, and brought into the clinic within 24 h. In the clinic, they were stored at 4 °C and processed within 24 h in the following manner: two sterile cotton swabs were inserted into the stool at three separate locations and then stored at −80 °C for DNA extraction. Approximately 1 g of stool was mixed with PBS buffer containing 10% glycerol and stored at −80 °C prior to use for phage counts. Remaining stool was transferred to a 100 mL sterile plastic container and stored at −80 °C for possible future analyses.

### 2.6. Phage Enumeration

Fecal samples stored in glycerol were thawed at room temperature. Samples were vortexed for 5 min with ceramic beads in 1 mL 2× sodium chloride magnesium sulfide (SM) buffer to homogenize [11]. A 1.5 mL aliquot was removed and centrifuged at 2000× *g* for 2 min, and supernatant was removed and centrifuged again at 140,000 rpm for 10 min. An amount of 500 uL of prepared sample was mixed with 100 uL of *E. coli* K12 culture (grown overnight in Luria Bertani (LB) broth on a 37 °C shaker to OD_600_ = 0.7 − 1.0) and incubated at 37 °C for 5 min. An amount of 3 mL of freshly prepared LB soft agar was then added to the mixture and immediately poured on LB agar plates which were pre-warmed to 37 °C for 30 min. Each sample was plated in duplicate and quantified using plated phage standard dilution plates.

### 2.7. Microbiota Assessment

Fecal DNA was extracted using the FastDNA^®^ Kit (MP Biomedicals, #116540400) following manufacturer’s protocol. The V4 region of the 16S rRNA gene was amplified following the Earth Microbiome Project protocol using the 515F-806R primer set [12] containing a unique 12 bp error correcting barcode included on the forward primer. Cycling and sequencing conditions were as previously described [13]. DNA extraction controls, no template PCR controls, and the Zymo mock community were included on each sequencing plate. Sequence reads were imported into QIIME2 version 2020.2 for analysis [14]. Briefly, the sequence reads were demultiplexed, and concatenated. Utilizing a Phred score cutoff of 30, upon examining the demultiplexed data, the reverse reads did not meet the quality filtering parameter. As such analysis proceeded with single-end sequences which were trimmed to 209 base pairs based on the same quality score. Reads were binned into ASVs using the DADA2 pipeline [15]. Taxonomic assignments were made using GreenGenes version 13.8. Samples with low reads or suspected contamination were removed and mitochondrial and chloroplast sequences were filtered from remaining samples. The resulting feature tables and taxonomy files were imported into MyPhyloDB version v.1.2.0 [16]. Alpha-diversity was calculated using Faith’s and Shannon metrics through the QIIME2 diversity plugin. Beta diversity was determined by Bray Curtis distance measurements and visualized by Principle Coordinates Analysis (PCoA) in MyPhyloDB. Differences in taxa among treatments were assessed in MyPhyloDB using univariate Analysis of Covariance (ANCoVA) and multivariate DiffAbund.

### 2.8. Statistical Analysis

Statistical analysis of clinical parameters (GI questionnaires, stool logs, metabolic panel analytes, dietary intakes, anthropometrics) and qPCR data was completed using GraphPad Prism, version 8.3.0. All data were analyzed using a 2-way mixed-model ANOVA with Sidak’s post hoc for multiple comparisons. In addition, baseline adjusted final values were analyzed using a 1-way ANOVA with Tukey’s post hoc for multiple comparisons. Statistical significance was assigned as *p* < 0.05 and a statistical trend was defined as between *p* < 0.10–0.051. Microbiota alpha-diversity parameters were analyzed using a one-way ANOVA with a non-parametric Kruskal–Wallis post hoc test, and β-diversity was analyzed by PERMANOVA with 1000 iterations. Analysis of Covariance (ANCoVA) with Tukey’s post-hoc for multiple comparisons and a negative binomial general linear model [17] were used to determine statistically different taxa between time points and treatment groups.

## 3. Results

### 3.1. Participant Characteristics

**Figure 1:**
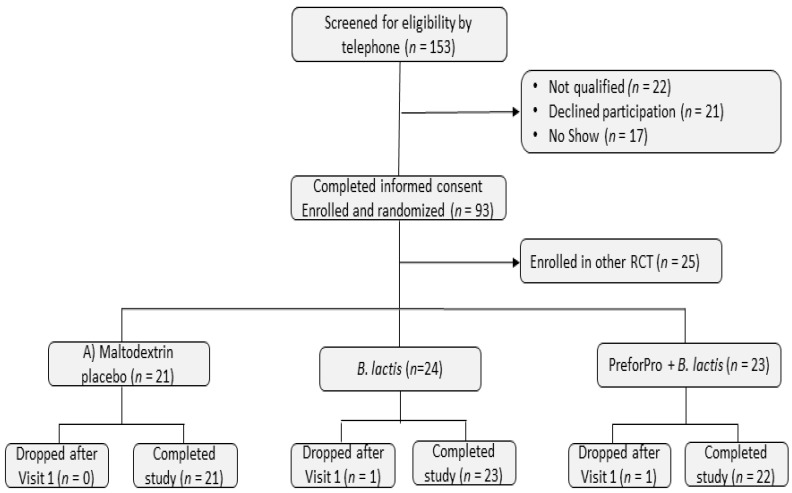
Consort flow diagram of participants through study enrollment to completion.

A total of 153 individuals were screened for eligibility. From this sample, 93 were eligible and enrolled in the study. Among these, 25 participants were enrolled in a different RCT with the same inclusion and exclusion protocol (Trotter et al., 2020, in press). Of the 68 enrolled in this branch of the study, two dropped after visit 1. A total of 66 individuals who met all the inclusion and exclusion criteria completed this 4-week, randomized, parallel-arm, double-blind, placebo-controlled clinical trial (Figure 1).

Approximately 12% of participants involved in the study experienced adverse events, with the severity of reported adverse events either mild or moderate. No adverse events were reported as severe or life-threatening, suggesting that there was a low risk associated with both treatments, and no participants dropped out of the study due to adverse events. The *B. lactis* BL04 treatment group experienced the highest incidence of adverse events, accounting for about 7.5% of the total reported study-related events. Major treatment-associated adverse events included constipation, bloating, flatulence, fatigue/lack of energy, or other.

### 3.2. Study Compliance

Overall compliance with the treatment protocol was about 95% with individual compliance ranging from 73–100%. Under the intention-to-treat analysis principle, all individual data were analyzed, regardless of compliance. Within treatment groups compliance was as follows: Placebo: average compliance = 97%, compliance range = 77–100%; *B. lactis* BL04: average compliance = 95%, compliance range = 80–100%; *B. lactis* BL04 + PreforPro: average compliance = 95%, compliance range = 73–100%.

The overall number of participants that completed the self-reported 24-h food recalls was low, with only about 53% of participants completing two dietary records at both baseline and during the 4-week intervention. Within each of the treatment groups, dietary compliance for completing the food recall was as follows: Placebo: average compliance = 48%, *B. lactis* BL04: average compliance = 58%, *B. lactis* BL04 + PreforPro: average compliance = 57%. From the dietary information collected, there were no significant differences in total calories, macronutrients, fiber, cholesterol, or saturated fat consumed over the course of the study within or between any of the treatment groups (Table 4). Finally, variability among and within treatment groups had no influence on body weight during the study as the average BMI was about 24 kg/m^2^ (see Table 1) for all groups both pre- and post-treatment and average weights fluctuated by <1 kg in each treatment group. Likewise, average values for analytes measured by the Metlyte plus CRP Reagent Disk did not vary significantly across groups or pre- to post-treatment within groups and all values remained within clinically normal ranges throughout the study (Supplemental Table S1).

### 3.3. GI Health and Digestion

**Figure 2:**
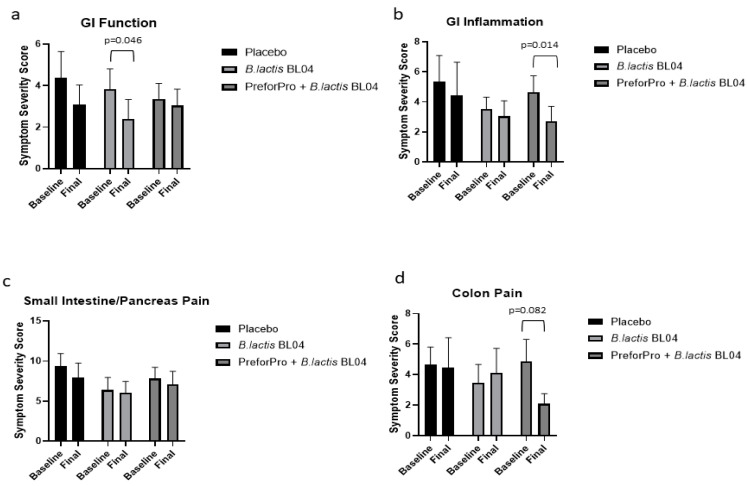
Average symptom severity before and after treatment as determined by gastrointestinal (GI) questionnaire. Average scores at baseline and after 4-weeks are shown for functional assessments of (**a**) Gastrointestinal function; (**b**) gastrointestinal inflammation; (**c**) small intestine pain; and (**d**) colon pain.

Total point scores for each section of the digestive health questionnaire represent symptom severity related to gastric function, GI inflammation, small intestine and pancreas pain, and colon pain. Changes in individual scores, for each section, pre- to post-treatment are shown in Supplemental Figure S1. There was a significant main effect for Time (*p* = 0.004) and average scores from the gastric function section of the questionnaire were significantly reduced (improved) with *B. lactis* BL04 treatment (Figure 2a; *p* = 0.046; [CI: −2.851 to −0.019]); although there were no significant changes in gastric function noted within the placebo or the PreforPro + *B. lactis* BL04 group or among any of the treatments. There was also a significant main effect for time (*p* = 0.005) in perceived improvement in GI inflammation and a significant reduction in the average symptom severity with the *B. lactis* BL04 + PreforPro treatment (Figure 2b; *p* = 0.005; [CI: −3.509 to −0.307]). While there were no other statistically significant differences observed, there was also a trend for improved colon pain in individuals on the *B. lactis* BL04 + PreforPro treatment (Figure 2d; *p* = 0.082; [CI: −5.802 to 0.2568]). Notably, while there were no other significant improvements, there were also no significant worsening of symptoms, indicating that both treatments, at the levels administered, were tolerable.

When comparing baseline adjusted (final-baseline) means among groups, there were no significant differences (Supplemental Figure S2). However, there was a small positive effect, determined using Cohen’s *d,* in symptom severity for the GI Inflammation and colon pain sections with *B. lactis* BL04 + PreforPro when compared to the placebo, while the effect of *B. lactis* alone was negligible (Table 5, [18]).

**Figure 3:**
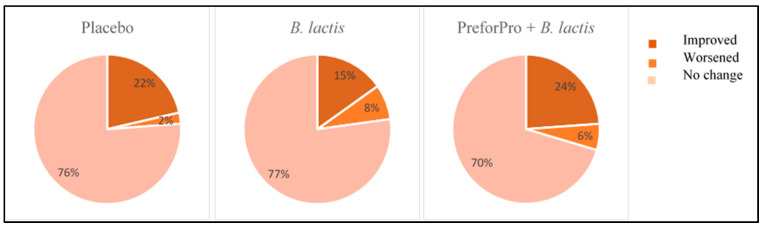
Priority changes in overall digestive symptoms from baseline to follow-up.

The GI health questionnaire scores corresponded to priority levels of mild (0–24 total points), moderate (25–48 total points), or severe (49–280 total points) symptoms in each of the target areas. Using these priority scores, we were able to calculate whether participants’ symptoms qualitatively worsened, improved, or stayed the same during the study (Figure 3). Regardless of the treatment group, most participants experienced no change in the overall priority level of their perceived GI distress. Only a small percentage of participants experienced an overall worsening of priority status from baseline to follow-up, with the lowest percentage of participants in the placebo group (2%) and the highest percentage in the *B. lactis* group (8%) moving from a lower to a higher priority score. The PreforPro + *B. lactis* group saw the greatest percentage (24%) of participants with overall improvements in the priority status of their symptoms (i.e., moved from a higher to a lower priority ranking). In comparison, only 15% of participants on *B. lactis* alone moved to a lower priority score after treatment.

The percentage of persons whose ratio of normal (Bristol Type 3, 4 or soft stool) to abnormal stool (Bristol Type 1, 2, 5, 6 or hard or watery stool) that improved, declined, or stayed the same is shown in Table 6. A decline in stool consistency was defined as having a higher proportion of bowel movements that were considered abnormal (hard or diarrhea) to stool that was normal (soft). The majority of participants experienced no change in stool consistency from week 1 to week 4. However, a substantial percentage of participants in the *B. lactis* BL04 (22%) and *B. lactis* BL04 + PreforPro (32%) groups experienced a decline in stool consistency from week 1 to week 4. Almost half of those in the *B. lactis* BL04 + PreforPro group saw no change in stool consistency from baseline to follow-up (41%) while the *B. lactis* BL04 group saw the greatest improvement in stool consistency over time (39%).

Because participants began taking their respective treatments concurrent with recording bowel movements, there may have been rapid changes in stool consistency that occurred within the first week of treatment, obscuring treatment effects. Therefore, we also calculated changes in stool consistency using only the first two recorded days compared to the reported stool consistency at week 4. We saw higher percentages of participants reporting improved stool consistency in both the Placebo and *B. lactis* BL04 groups (33% and 52%, respectively). Most participants in the *B. lactis* BL04 + PreforPro group had a decline in stool consistency (59%) from baseline to follow-up. There was also a noticeable negative placebo effect with 48% of participants reporting a decline in stool consistency in the placebo group.

### 3.4. Microbiota Changes

Phage plating was conducted to confirm the presence of viable *E. coli*-targeting phages in stool samples. No phages were detected in baseline samples from any treatment group or from the 4-week samples collected from individuals in the Placebo or the *B. lactis* BL04 groups. About 44% of the final samples collected from individuals assigned to the *B. lactis* BL04 + PreforPro group had viable phages detected, although the total number recovered varied from 7–30,531 pfu/gram with the average being 5103 pfu/gram.

**Figure 4:**
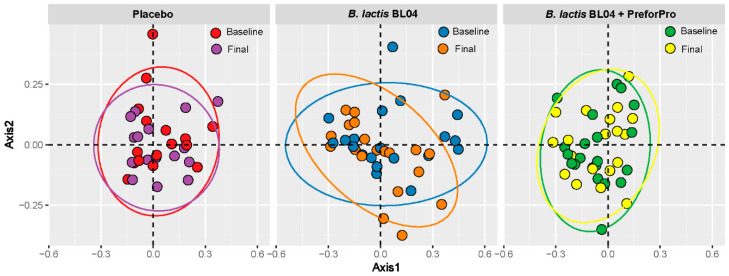
Principle Coordinates Analysis (PCoA) of Bray–Curtis distances at baseline and final visit for each treatment group.

Using 16s rRNA amplicon sequencing, we assessed whether the three treatments impacted the gut microbial community. The microbiota in all samples was primarily represented by the phyla Actinobacteria, Bacteroidetes, Firmicutes, Proteobacteria, and Verrucomicrobiota and the proportion of these phyla did not significantly differ between groups or over time. The alpha-diversity metrics for Faith’s Phylogenetic Diversity (Faith’s PD), Shannon’s diversity, and Pielou’s Evenness did not significantly differ between treatment groups ar within any treatment group over time. (Supplemental Table S2). Likewise, examining β-diversity using Bray–Curtis distances visualized by PCoA with Non-Metric Dimensional Scaling (NMDS) revealed no significant clustering by treatment, as determined by PERMANOVA (Figure 4).

**Figure 5:**
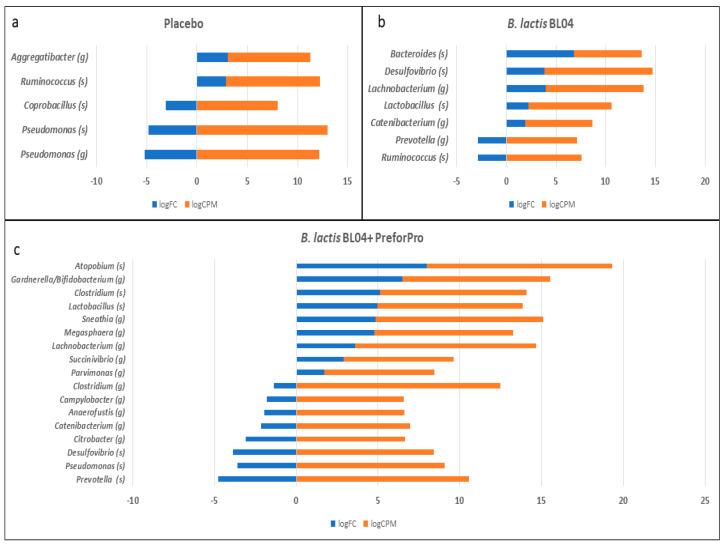
Changes in specific taxa within treatment groups, identified using a gene-wise negative binomial generalized linear model (EdgeR). (**a**) Placebo (**b**) lactis BL04 (**c**) lactis BL04+PreforPro, Log Fold Change = logFC; log Count Per Million = logCPM.

Although no changes were observed in the overall composition and diversity of the microbiota, some select taxa were significantly increased or decreased in the two probiotic treatment groups, but not in the Placebo. Using the DiffAbund function in MyPhyloDB, we saw some changes in specific taxa. Figure 5 shows the baseline to final visit changes within each treatment group with the blue bars representing log fold-change (logFC) and the orange bars representing the log count per million (logCPM). Within the placebo group, there were decreases in *Pseudomonas* and *Coprobacillus* and increases in *Aggregatibacter* and *Ruminococcus*. This increase in *Ruminococcus (gnavus)* was also observed using a less sensitive parametric ANCoVA analysis (*p* = 0.04). *Lachnobacterium* and *Lactobacillus* increases were noted with both *B. lactis* treatment groups; however, the increase in *Lachnobacterium* was approximately equal between these groups while there was a ~6-fold (3-logFC) increase in *Lactobacillus* observed in *B. lactis* BL04 + PreforPro compared to the probiotic alone. We also saw increases in *Atopobium*, *Gardnerella/Bifidobacterium*, and *Clostridium* in the group consuming phages. The increase in *Clostridium (citroniae*) was also observed in this treatment group when analyzing with ANCoVA (*p* = 0.03). Decreases were noted in *Citrobacter* and *Desulfovibrio*, which are taxa often associated with increased gut inflammation and GI disorders [19,20]. *Prevotella* was concurrently decreased with both groups receiving probiotic treatments, although the *B. lactis* BL04 + PreforPro showed a slightly greater decrease. Finally, opposing changes in *Catenibacterium* and *Desulfovibrio* were observed between these treatment groups.

Additionally, we queried our sequence data for reads that matched *E. coli* in BLASTn to determine whether phage consumption altered levels of this taxa. We found several ASV’s that matched *E. coli*, but which could not be confirmed because of a high degree of homology in the sequenced region, resulting in equal matches with several closely related taxa. However, an assessment of these putative *E. coli* reads revealed a trending decrease (ANCoVA; *p* = 0.094) from baseline to the final visit with *B. lactis* BL04 + PreforPro, which was not observed in the other treatment groups (Supplemental Figure S3).

## 4. Discussion

There is growing interest in the incorporation of phages with probiotic dietary supplements. Phages can target specific pro-inflammatory or pathogenic organisms in the gut and potentially enhance the GI benefits of the probiotics, although evidence for these effects in human trials is currently lacking. In this randomized, double-blind, placebo-controlled clinical study, we observed the effects of *B. lactis* BL04, with or without the *E.coli*-targeting phage mixture marketed as PreforPro, on gastrointestinal well-being and modulation of the gut microbiota. The beneficial effect of supplementation with *B. lactis* BL04 appeared restricted to improvements in symptoms related to gastric function and stool consistency in 52% of participants. Participants who consumed *B. lactis* BL04 + PreforPro showed improvements in digestive symptoms related to GI inflammation and colon pain and had the highest percentage of individuals reporting overall reduced symptom severity. This is largely in agreement with a previous study, where Ginden et al. reported that PreforPro alone resulted in an improvement in gastric function and colon pain, but likewise reported a placebo effect [9]. Additionally, in the population of phage-only consuming participants, a higher percentage of participants reported an overall worsening in symptoms than was reported in the current study. This indicates that there might be some additive benefit to consuming these phages with a probiotic. However, the probiotic used in this study, *B. lactis* BL04 alone, elicited minimal effects on our study population, which was largely healthy adults with no diagnosed GI diseases. *Bifidobacterium lactis* BL04 is often studied in conjunction with other probiotic strains, and a recent study suggested that a five-strain mixture, containing *B. lactis* BL04, improved symptoms of antibiotic-associated diarrhea when given at a high dose (>10^10^ CFU) [21]. Our study used a single strain, lower dose (10^9^ CFU), and tested on a healthy population. While most people occasionally experience GI distress or irregular bowel movements, it is likely the effects of this probiotic are more subtle in healthy individuals. It would be interesting to determine whether the benefits we observed are more pronounced with a combination of probiotic and/or in patients with GI disorders.

Consistent with other published phage literature, the combination of *B. lactis* BL04 + PreforPro did not significantly impact gut ecology, with no significant shifts in either alpha- or beta-diversity parameters or large phyla-level changes in taxa. This concurs with our previous study, which looked at the impacts of the phages alone on gut microbiota [10]. Similarly, *E. coli*-targeting phages added to a simulated gut environment with a defined microbial consortium only showed effects on the target species and did not disrupt commensal organisms [22]. However, various lytic phages administered to gnotobiotic mice transplanted with a defined human commensal microbiota did demonstrate cascading effects on many of the non-target species, likely mediated by complex ecological interactions between bacteria [23]. This consortium was made up of only 10 bacterial species, suggesting that effects on these species may have been more profound than if they were observed within the context of an intact human gut containing hundreds of bacterial species.

Despite a lack of global alterations in the microbiota, the treatments were associated with changes in a small number of taxa. Most notably, we saw an increase in taxa identified as *Lactobacillus* after 4-weeks of both *B. lactis* BL04 and *B. lactis* BL04 + PreforPro. However, the increase was 10-fold greater than baseline levels when phages were included in the *B. lactis* treatment compared to a ~4-fold increase seen with the probiotic alone. Oral administration of a lytic phage induced similar increases in *Lactobacillus* in a mouse model, as well as increasing *Bifidobacterium* [24]. It is interesting to note that we did not observe increased *Bifidobacterium* in either treatment group, despite daily consumption of 10^9^ CFU of *B. lactis* BL04. A similar finding was reported in a study of healthy volunteers taking Amoxicillin and a multi-species probiotic, which included *B. lactis*, *E. faecalis* and several other *Bifidobacteria* and *Lactobacilli*. Using culture-based methods, the investigators only reported an increase in stool *Enterococci* [25]. We used a sequencing approach, which is not sufficiently sensitive to distinguish bacteria at the species level and it is possible that the baseline levels of *Bifidobacterium* across our treatment groups were sufficiently high to obscure any treatment effects. Finally, there were several taxa that have demonstrated inflammatory effects on the gut that were reduced after treatment of *B. lactis* BL04 + PreforPro. Specifically, there were decreases in *Citrobacter* and *Desulfovibrio. Citrobacter* overgrowth is associated with bloating, and both Faber et al. [26] and Gangi et al. [19] reported increased levels of *Citrobacter* in IBS patients. In the Faber study, symptoms of bloating and abdominal pain were alleviated after reducing *Citrobacter,* and other pro-inflammatory bacteria and yeasts, by antibiotic treatment and supplementation with probiotics [26]. *Desulfovibrio* is a sulfate-reducing bacteria that is also often associated with IBS [20] and induces alterations in colonic architecture, cellular infiltration into the lamina propria, and increased inflammatory immune responses in mouse models of colitis [27]. Therefore, the reduction of these bacteria may have been responsible for the symptom reduction reported in our participant population and suggest that future trials in a population with IBS or colitis may be warranted.

The study has some strengths and limitations worth considering. One major strength is that this study is a randomized, double-blind, placebo-controlled clinical trial. Randomized controlled trials are the gold standard for study design since they eliminate much inherent bias that other study designs introduce. Similarly, compliance across treatments was high at about 95% with no significant dietary differences in total calories, macronutrients, fiber, cholesterol, or saturated fat within any of the treatment groups. However, we recognize that there are several limitations to this study as well. First, although we saw pre- to post-treatment improvements with treatment in several of the GI parameters, there were no significant differences in score change across groups and the effect size, while significant, was small. This may be due to the fact that the study was powered from previously collected data [10] to detect differences in the detection of *Bifidobacterium* populations among groups but was not specifically powered for the GI outcomes. Therefore, the study may not have been sufficiently powered to detect these differences between treatment groups. Another limitation was that true baseline data are lacking for stool consistency measurements, as these were recorded concurrently with the start of the study protocol. To minimize the impact of treatment effects on stool consistency measures, we analyzed Day 1 and 2 as a baseline in comparison to Week 4 in addition to comparing data from Week 1 to Week 4. Another limitation is that participants had the option of numerically assessing their stool using the Bristol Stool Chart or provide a designation of hard (H), soft (S), or diarrhea (D). This mixture in data capture methods prevented the quantitative assessment of stool changes, although it was still useful for functional assessment. Finally, the high participant burden of recording every bowel movement for a month could have led to participants not recording all of their stools or recording only their first bowel movement of the day.

Another limitation of the study was our ability to detect the phages in stool samples post-consumption. Due to blinding during the study, all collected samples were plated for phage enumeration. While no baseline samples, nor any in the probiotic alone and placebo group samples resulted in plaque detection, only about 40% of the samples from the group receiving the bacteriophage cocktail resulted in plaque formation after incubation with *E. coli*. There are several possibilities for the low detection of phages in these samples, including both technical challenges and individual participant factors. First, in order to reduce variability due to plating and enumeration techniques, samples were frozen and then batch processed at the end of the study, which may have resulted in a loss of viability, particularly in samples stored for longer periods. In addition, stool samples are heterogenous and few studies have looked at the distribution of phages within stool, so it is possible that our subsample-based approach was insufficient to detect low populations of phages in the stool. Finally, a study looking at the survival of phages in a simulated digestive tract found that meal composition, pH and other factors affected phage survival and detection [28]. Therefore, it is likely that the timing of capsule consumption, the pH of the individual’s GI tract, and the meal composition prior to sample collection all may have played a role in the survival of phages in the stool. This raises some interesting possibilities for future research in terms of looking at the timing and delivery of phages, as well as interindividual variability in efficacy and stool pH to determine the optimal therapeutic conditions for phage supplementation.

In conclusion, the present study demonstrates that oral supplementation of *B. lactis* BL04 over the course of 4 weeks has the potential to improve stool consistency, and *B. lactis* BL04 with PreforPro can significantly improve some GI symptoms. In addition, phage consumption did not significantly disrupt the gut microbial community but was associated with an increased relative abundance of some beneficial species, like *Lactobacillus*, and decreases in certain pro-inflammatory taxa. While we did not observe significant improvements in all aspects of GI well-being or stool consistency, the findings of this study indicate a low risk for oral supplementation with phages and suggest that probiotic taken with a phage cocktail may offer a safe solution for the management of occasional GI symptoms in healthy adults.

## Figures and Tables

**Table 1:** Participant inclusion and exclusion criteria.

Inclusion Criteria	Exclusion Criteria
Men and women	Pregnant and breastfeeding women
Aged 18–65 years	Taking medication that would influence the endpoints of the study (statins, metformin, nonsteroidal anti-inflammatory drugs, monoamine oxidase inhibitors, blood pressure medications) and taking probiotics and/or botanical supplements that target the GI tract or gut microbiota.
Normal, overweight, or class 1 obese (BMI 20–34.9 kg/m^2^)	Current diagnosis of cancer, liver or kidney disease, gastrointestinal diseases, and metabolic disorders.
	Antibiotic use within the 2 months prior to enrollment.

**Table 2:** Participant baseline characteristics.

Treatment	Male (*n*)	Female (*n*)	Height (cm)	Weight (kg)	BMI	Age
Placebo	8	13	169.9 ± 8.2	71.3 ± 10.6	24.67 ± 2.8	36.5 ± 13.0
*B. lactis* BL04	10	13	169.9± 8.2	71.5 ± 10.7	24.7 ± 2.8	36.5 ± 13.2
*B. lactis* BL04 + PreforPro	7	15	170.1 ± 8.22	71.6 ± 10.4	24.7 ± 2.71	36.1 ± 13.3

Data represent mean ± SD.

**Table 3:** Priority status based on scores from the digestive health questionnaire.

		Questionnaire Section			
		Gastric Function	GI Inflammation	SI & Pancreas	Colon
Symptom Severity	Low	1–4	1–4	2–8	2–8
	Moderate	5–8	5–8	9–16	9–16
	High	9–56	9–72	17–80	17–72

**Abbreviations:** GI, gastrointestinal; SI, small intestine.

**Table 4:** Self-reported dietary intake.

	Placebo (*n* = 10)		*B. lactis* BL04 (*n* = 14)		*B. lactis* BL04 + PreforPro (*n* = 12)	
	Baseline	Final	Baseline	Final	Baseline	Final
Energy (KCAL)	2269 ± 999	2089 ± 982	1775 ± 735	1853 ± 675	1739 ± 474	1905 ± 719
PRO (g)	99 ± 50	96 ± 50	85 ± 49	74 ± 29	70 ± 18	68 ± 27
TFAT (g)	103 ± 56	86 ± 42	73 ± 27	84 ± 44	66 ± 30	73 ± 29
SFAT(g)	33 ± 18	27 ± 14	21 ± 9	29 ± 21	19 ± 6	23 ± 12
CHOL (mg)	358 ± 259	324 ± 271	282 ± 299	277 ± 281	215 ± 101	180 ± 129
CARB (g)	240 ± 104	225 ± 118	194 ± 103	199 ± 91	209 ± 54	222 ± 71
FIBER (g)	25 ± 11	22 ± 12	27 ± 19	23 ± 16	26 ± 21	24 ± 14

Data represent mean ± SD. No values were statistically significant with a *p*-value < 0.05. Abbreviations: PRO (protein), TFAT (total fat), SFAT (saturated fat), CHOL (cholesterol), CARB (carbohydrates).

**Table 5:** Effect size (Cohen’s *d*) of treatments relative to the placebo.

	Gastric Function	GI Inflammation	SI and Pancreas Pain	Colon Pain
*B. lactis* BL04	0.05	−0.13	−0.17	−0.14
*B. lactis* BL04 + PreforPro	−0.38	0.30	0.11	0.45

**Table 6:** Stool consistency changes from baseline to follow-up visit.

	Stool Changes between Week 1 and Week 4			Stool Changes between Days 1 and 2 and Week 4		
	Improved	Declined	Same	Improved	Declined	Same
Placebo	29%	33%	38%	33%	48%	19%
*B. lactis* BL04	39%	22%	39%	52%	39%	9%
*B. lactis* BL04 + PreforPro	27%	32%	41%	27%	59%	14%

No values were found to be statistically different.

## Supplementary Materials

The following are available online at https://www.mdpi.com/2072-6643/12/8/2474/s1, Figure S1: Individual scores at baseline and post-treatment for each of the 4 sections of the gastrointestinal health questionnaire. Figure S2: Baseline adjusted scores for each section of the gastrointestinal health questionnaire across treatments. Figure S3: Abundance of unclassified Enterobacteriaceae that putatively belong to *Escherichia coli.* Table S1: Metabolic parameters in blood before and after treatment for each group. Table S2: Alpha Diversity Metrics. File S1: Gastrointestinal (GI) Health Assessment.

## References

[1] Fava F., Rizzetto L., Tuohy K.M. (2018). Gut microbiota and health: Connecting actors across the metabolic system. Proc. Nutr. Soc..

[2] Ianiro G., Murri R., Sciumè G.D., Impagnatiello M., Masucci L., Ford A.C., Law G.R., Tilg H., Sanguinetti M., Cauda R. (2019). Incidence of bloodstream infections, length of hospital stay, and survival in patients with recurrent clostridioides difficile infection treated with fecal microbiota transplantation or antibiotics a prospective cohort study. Ann. Intern. Med..

[3] Digestive Health Supplements Market Size|Industry Report, 2019–2025. https://www.grandviewresearch.com/industry-analysis/digestive-health-supplements-market.

[4] Sanders M.E., Benson A., Lebeer S., Merenstein D., Klaenhammer T.R. (2018). Shared mechanisms among probiotic taxa: Implications for general probiotic claims. Curr. Opin. Biotechnol..

[5] Goldin B., Gorbach S.L. (2008). Clinical Indications for Probiotics: An Overview. Clin. Infect. Dis..

[6] Ritchie M.L., Romanuk T.N. (2012). A Meta-Analysis of Probiotic Efficacy for Gastrointestinal Diseases. PLoS ONE.

[7] Zhang C., Jiang J., Tian F., Zhao J., Zhang H., Zhai Q., Chen W. (2020). Meta-analysis of randomized controlled trials of the effects of probiotics on functional constipation in adults. Clin. Nutr..

[8] Hungin A.P.S., Mulligan C., Pot B., Whorwell P., Agréus L., Fracasso P., Lionis C., Mendive J., De Foy J.-M.P., Rubin G. (2013). Systematic review: Probiotics in the management of lower gastrointestinal symptoms in clinical practice—An evidence-based international guide. Aliment. Pharmacol. Ther..

[9] Gindin M., Febvre H.P., Rao S., Wallace T.C., Weir T.L. (2019). Bacteriophage for Gastrointestinal Health (PHAGE) Study: Evaluating the Safety and Tolerability of Supplemental Bacteriophage Consumption. J. Am. Coll. Nutr..

[10] Febvre H.P., Rao S., Gindin M., Goodwin N.D.M., Finer E., Vivanco J.S., Lu S., Manter D.K., Wallace T.C., Weir T.L. (2019). PHAGE Study: Effects of Supplemental Bacteriophage Intake on Inflammation and Gut Microbiota in Healthy Adults. Nutrients.

[11] Castro-Mejía J., Muhammed M.K., Kot W., Neve H., Franz C.M.A.P., Hansen L.H., Vogensen F.K., Nielsen D.S. (2015). Optimizing protocols for extraction of bacteriophages prior to metagenomic analyses of phage communities in the human gut. Microbiome.

[12] Caporaso J.G., Lauber C.L., Walters W.A., Berg-Lyons D., Huntley J., Fierer N., Owens S., Betley J., Fraser L., Bauer M. (2012). Ultra-high-throughput microbial community analysis on the Illumina HiSeq and MiSeq platforms. ISME J..

[13] Lee D.M., E Ecton K., Trikha S.R.J., Wrigley S.D., Thomas K.N., Battson M.L., Wei Y., Johnson S.A., Weir T.L., Gentile C.L. (2020). Microbial Metabolite Indole-3-Propionic Acid Supplementation Does Not Protect Mice from the Cardiometabolic Consequences of a Western Diet. Am. J. Physiol. Gastroint. Liver Physiol..

[14] Bolyen E., Rideout J.R., Dillon M.R., Bokulich N.A., Abnet C.C., Al-Ghalith G.A., Alexander H., Alm E.J., Arumugam M., Asnicar F. (2019). Reproducible, interactive, scalable and extensible microbiome data science using QIIME 2. Nat. Biotechnol..

[15] Callahan B.J., McMurdie P.J., Rosen M.J., Han A.W., Johnson A.J.A., Holmes S.P. (2016). DADA2: High-resolution sample inference from Illumina amplicon data. Nat. Methods.

[16] Manter D.K., Korsa M., Tebbe C., Delgado J.A. (2016). myPhyloDB: A local web server for the storage and analysis of metagenomic data. Database.

[17] Chen Y., McCarthy D., Ritchie D., Robinson M., Smyth G., Hall E. Edger: Differential Analysis of Sequence Read Count Data User’s Guide. https://bioconductor.riken.jp/packages/release/bioc/vignettes/edgeR/inst/doc/edgeRUsersGuide.pdf.

[18] Cohen J. Statistical Power Analysis for the Behavioral Sciences Second Edition. http://www.utstat.toronto.edu/~brunner/oldclass/378f16/readings/CohenPower.pdf.

[19] Ganji L., Alebouyeh M., Shirazi M.H., Eshraghi S.S., Mirshafiey A., Daryani N.E., Zali M.R. (2016). Dysbiosis of fecal microbiota and high frequency of Citrobacter, Klebsiella spp., and Actinomycetes in patients with irritable bowel syndrome and gastroenteritis. Gastroenterol. Hepatol. Bed Bench.

[20] Loubinoux J., Bronowicki J.P., Pereira I.A., Mougenel J.L., Le Faou A.E. (2002). Sulfate-reducing bacteria in human feces and their association with inflammatory bowel diseases. FEMS Microbiol. Ecol..

[21] Ouwehand A.C., Donglian C., Weijian X., Stewart M., Ni J., Stewart T., Miller L.E. (2014). Probiotics reduce symptoms of antibiotic use in a hospital setting: A randomized dose response study. Vaccine.

[22] Cieplak T., Soffer N., Sulakvelidze A., Nielsen D.S. (2018). A bacteriophage cocktail targeting *Escherichia coli* reduces *E. coli* in simulated gut conditions, while preserving a non-targeted representative commensal normal microbiota. Gut Microbes.

[23] Hsu B.B., Gibson T.E., Yeliseyev V., Liu Q., Lyon L., Bry L., A Silver P., Gerber G.K. (2019). Dynamic Modulation of the Gut Microbiota and Metabolome by Bacteriophages in a Mouse Model. Cell Host Microbe.

[24] Bao H.-D., Pang M.-D., Olaniran A., Zhang X.-H., Zhang H., Zhou Y., Sun L.-C., Schmidt S., Wang R. (2018). Alterations in the diversity and composition of mice gut microbiota by lytic or temperate gut phage treatment. Appl. Microbiol. Biotechnol..

[25] Koning C.J.M., A E Jonkers D.M., E Stobberingh E., Mulder L., Rombouts F.M., Stockbrügger R.W. (2008). The Effect of a Multispecies Probiotic on the Intestinal Microbiota and Bowel Movements in Healthy Volunteers Taking the Antibiotic Amoxycillin. Am. J. Gastroenterol..

[26] Faber S.M. (2000). Treatment of abnormal gut flora improves symptoms in patients with irritable bowel syndrome. Am. J. Gastroenterol..

[27] Figliuolo V.R., Dos Santos L.M., Abalo A., Nanini H.F., Santos A., Brittes N.M., Bernardazzi C., Souza H., Vieira L.Q., Coutinho-Silva R. (2017). Sulfate-reducing bacteria stimulate gut immune responses and contribute to inflammation in experimental colitis. Life Sci..

[28] Samtlebe M., Denis S., Chalancon S., Atamer Z., Wagner N., Neve H., Franz C., Schmidt H., Blanquet-Diot S., Hinrichs J. (2018). Bacteriophages as modulator for the human gut microbiota: Release from dairy food systems and survival in a dynamic human gastrointestinal model. LWT.

