# Supplementary material for: PHAGE-2 Study: Supplemental Bacteriophages Extend Bifidobacterium animalis subsp. lactis BL04 Benefits on Gut Health and Microbiota in Healthy Adults
Source: Nutrients. 2020 Aug 17;12(8):2474. doi: 10.3390/nu12082474 (PMC7468981; doi:10.3390/nu12082474)
Supplement: Supplementary file 1 [file nutrients-12-02474-s001.zip › supplementary-1.docx]

**Supplemental Tables**

**Supplemental Table 1.** Metabolic parameters in blood before and after treatment for each group.

|  | Placebo  (*n* = 16) | | *B. lactis* BL04  (*n* = 20) | | *B. lactis* BL04 + PreforPro  (*n* = 20) | |
| --- | --- | --- | --- | --- | --- | --- |
|  | **Baseline** | **Final** | **Baseline** | **Final** | **Baseline** | **Final** |
| Glucose | 88 ± 19 | 93 ± 9 | 91 ± 5 | 93 ± 5 | 92 ± 5 | 94 ± 6 |
| BUN | 12 ± 4 | 13 ± 3 | 12 ± 4 | 12 ± 3 | 12 ± 2 | 12 ± 3 |
| CRE | 0.8 ± 0.2 | 0.8 ± 0.2 | 0.9 ± 0.2 | 0.8 ± 0.2 | 0.8 ± 0.2 | 0.9 ± 0.2 |
| CK | 148 ± 47 | 161 ± 66 | 160 ± 75 | 180 ± 200 | 171 ± 142 | 140 ± 99 |
| Na+ | 138 ± 3 | 137 ± 3 | 139 ± 6 | 138 ± 3 | 137 ± 3 | 137 ± 3 |
| K+ | 4.5 ± 1.1 | 4.3 ± 0.4 | 4.8 ± 0.5 | 4.5 ± 0.4 | 4.6 ± 0.5 | 4.6 ± 0.5 |
| Cl^-^ | 107 ± 2 | 108 ± 2 | 107 ± 3 | 109 ± 3 | 107 ± 3 | 109 ± 3 |
| tCO2 | 26 ± 2 | 26 ± 1 | 25 ± 5 | 26 ± 2 | 25 ± 2 | 26 ± 2 |
| Data represent mean ± SD. No values were statistically significant with a *p*-value <0.05 Abbreviations: Blood Urea Nitrogen (BUN); Creatinine (CRE); Creatinine Kinase (CK); Sodium (NA); Potassium (K), Total Carbon Dioxide (tCO2) | | | | | | |

**Supplemental Table 2.** Alpha Diversity Metrics

|  | Placebo  (*n* = 16) | | *B. lactis* BL04  (*n* = 20) | | *B. lactis* BL04 +PreforPro (*n* = 20) | |
| --- | --- | --- | --- | --- | --- | --- |
|  | Baseline | Final | Baseline | Final | Baseline | Final |
| Pileu’s Evenness | 0.70 ± 0.05 | 0.71 ± 0.04 | 0.69 ± 0.08 | 0.68 ± 0.06 | 0.71 ± 0.07 | 0.70 ± 0.06 |
| Faith’s Phylogenetic Diversity | 11.15 ± 2.36 | 11.41 ± 1.98 | 10.86 ± 3.05 | 10.58 ± 2.405 | 11.11 ± 3.97 | 10.99 ± 3.54 |
| Shannon Diversity | 5.10 ± 0.50 | 5.22 ± 0.44 | 4.95 ± 0.81 | 4.89 ± 0.58 | 5.14 ± 0.78 | 5.12 ± 0.70 |
| Data represents mean ± SD. No values were statistically significant with a *p*-value < 0.05. | | | | | | |

**Supplemental Figures**


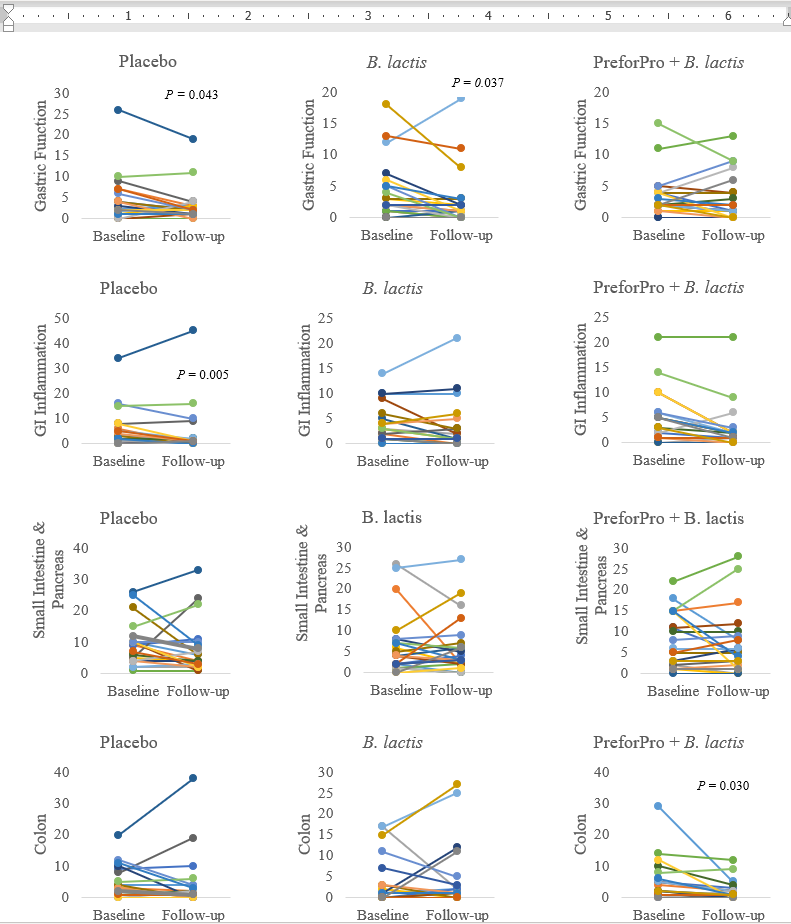


**Supplemental Figure 1.** Individual scores at baseline and 4 weeks post-treatment for each of the 4 sections of the gastrointestinal health questionnaire.

**
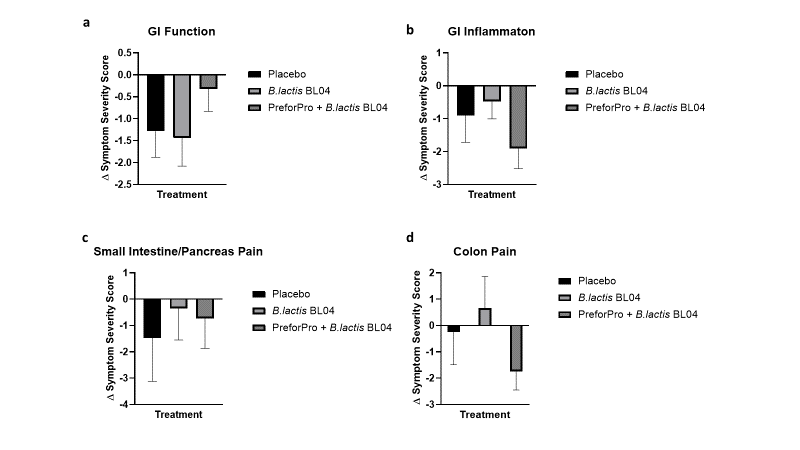
**

**Supplemental Figure 2.** Baseline adjusted scores for each section of the gastrointestinal health questionnaire across treatments. Data represent mean ± SEM. There were no significant differences in baseline adjusted scores across treatment groups (*p* = 0.05).

**Supplemental Figure 3.** Abundance of unclassified Enterobacteriaceae that putatively belong to *Escherichia coli.* Data represent mean ± SEM.
