# Supplementary material for: PHAGE-2 Study: Supplemental Bacteriophages Extend Bifidobacterium animalis subsp. lactis BL04 Benefits on Gut Health and Microbiota in Healthy Adults
Source: Nutrients. 2020 Aug 17;12(8):2474. doi: 10.3390/nu12082474 (PMC7468981; doi:10.3390/nu12082474)
Supplement: Supplementary file 1 [file nutrients-12-02474-s001.zip › supplementary-2-File S1.pdf]

Gastrointestinal (GI) Health Assessment

An estimated 70% of the body's immune cells reside in the GI tract, suggesting that maintaining GI health is essential to immune system function and overall health.

The GI system is quite complex, and evaluating GI health in order to recommend a therapeutic support program can be a difficult undertaking in a busy practice. To simplify matters, the following graph provides a quick scoring system for GI Health as assessed by the Health Appraisal Questionnaire (see reverse).

Health Appraisal Graph

		LOW PRIORITY				MODERATE PRIORITY				HIGH PRIORITY				Initial Test Score	Retest Score
SECTION A	Gastric Function	1	2	3	4	5	6	7	8	20	32	44	56		
SECTION B	GI Inflammation	1	2	3	4	5	6	7	8	24	40	56	72		
SECTION C	Small Intestine & Pancreas	2	4	6	8	10	12	14	16	32	48	64	80		
SECTION D	Colon	2	4	6	8	10	12	14	16	30	44	58	72		

© 2006 Lyra Heller, Michael Katke. Reproduction, photocopying, storage or transmission by magnetic or electronic means without permission is strictly prohibited by law.

HEALTH APPRAISAL QUESTIONNAIRE—PART I

Name ______________________________ **Date** ______________

DIRECTIONS

This questionnaire asks you to assess how you have been feeling **during the last four months**. This information will help you keep track of how your physical, mental and emotional states respond to changes you make in your eating habits, priorities, supplement program, social and family life, level of physical activity and time spent on personal growth. All information is held in strict confidence. Take all the time you need to complete this questionnaire.

For each question, circle the number that best describes your symptoms:

- **0 = No or Rarely**—You have never experienced the symptom or the symptom is familiar to you but you perceive it as insignificant (monthly or less)
- **1 = Occasionally**—Symptom comes and goes and is linked in your mind to stress, diet, fatigue or some identifiable trigger
- **4 = Often**—Symptom occurs 2-3 times per week and/or with a frequency that bothers you enough that you would like to do something about it
- **8 = Frequently**—Symptom occurs 4 or more times per week and/or you are aware of the symptom every day, or it occurs with regularity on a monthly or cyclical basis

Some questions require a YES or NO response: 0 = NO 8 = YES

SECTION A

	No/Rarely	Occasionally	Often	Frequently
1. Indigestion, food repeats on you after you eat	0	1	4	8
2. Excessive burping, belching and/or bloating following meals	0	1	4	8
3. Stomach spasms and cramping during or after eating	0	1	4	8
4. A sensation that food just sits in your stomach creating uncomfortable fullness, pressure and bloating during or after a meal	0	1	4	8
5. Bad taste in your mouth	0	1	4	8
6. Small amounts of food fill you up immediately	0	1	4	8
7. Skip meals or eat erratically because you have no appetite	0	1	4	8
**Total points**				

SECTION B

	No/Rarely	Occasionally	Often	Frequently
1. Strong emotions, or the thought or smell of food aggravates your stomach or makes it hurt	0	1	4	8
2. Feel hungry an hour or two after eating a good-sized meal	0	1	4	8
3. Stomach pain, burning and/or aching over a period of 1-4 hours after eating	0	1	4	8
4. Stomach pain, burning and/or aching relieved by eating food; drinking carbonated beverages, cream or milk; or taking antacids	0	1	4	8
5. Burning sensation in the lower part of your chest, especially when lying down or bending forward	0	1	4	8
6. Digestive problems that subside with rest and relaxation	(0)No			(8)Yes
7. Eating spicy and fatty (fried) foods, chocolate, coffee, alcohol, citrus or hot peppers causes your stomach to burn or ache	0	1	4	8
8. Feel a sense of nausea when you eat	0	1	4	8
9. Difficulty or pain when swallowing food or beverage	0	1	4	8
**Total points**				

SECTION C

	No/Rarely	Occasionally	Often	Frequently
1. When massaging under your rib cage *on your left side*, there is pain, tenderness or soreness	0	1	4	8
2. Indigestion, fullness or tension in your abdomen is delayed, occurring 2-4 hours after eating a meal	0	1	4	8
3. Lower abdominal discomfort is relieved with the passage of gas or with a bowel movement	0	1	4	8
4. Specific foods/beverages aggravate indigestion	0	1	4	8
5. The consistency or form of your stool changes (e.g., from narrow to loose) within the course of a day	0	1	4	8
6. Stool odor is embarrassing	0	1	4	8
7. Undigested food in your stool	0	1	4	8
8. Three or more large bowel movements daily	0	1	4	8
9. Diarrhea (frequent loose, watery stool)	0	1	4	8
10. Bowel movement shortly after eating (within 1 hour)	0	1	4	8
**Total points**				

SECTION D

	No/Rarely	Occasionally	Often	Frequently
1. Discomfort, pain or cramps in your colon (lower abdominal area)	0	1	4	8
2. Emotional stress and/or eating raw fruits and vegetables causes abdominal bloating, pain, cramps or gas	0	1	4	8
3. Generally constipated (or straining during bowel movements)	0	1	4	8
4. Stool is small, hard and dry	0	1	4	8
5. Pass mucus in your stool	0	1	4	8
6. Alternate between constipation and diarrhea	0	1	4	8
7. Rectal pain, itching or cramping	0	1	4	8
8. No urge to have a bowel movement	(0)No			(8)Yes
9. An almost continual need to have a bowel movement	(0)No			(8)Yes
**Total points**				
